# Comparison of Subclavian and Peripheral Intravenous Cannula Insertion in Critically Ill Patients Arriving in Emergency Department

**DOI:** 10.7759/cureus.5452

**Published:** 2019-08-21

**Authors:** Khalid Azam, Khurram Shahzad, Naima Anwar, Sadiq Zia

**Affiliations:** 1 Pulmonology, Combined Military Hospital, Lahore, PAK; 2 Cardiology, Combined Military Hospital, Lahore, PAK; 3 Neurosurgery, Lahore General Hospital, Lahore, PAK; 4 Medicine, Zia Hospital, Sadiqabad, PAK

**Keywords:** peripheral intravenous cannulation, pivc, subclavian vein cannula insertion, supraclavicular approach

## Abstract

Introduction: Peripheral intravenous cannulation (PIVC) is a universal procedure to get venous access in hospital emergency settings. Sometimes, for critically ill patients in an emergency department, when we cannot get peripheral venous access, a central venous access could be established by percutaneous subclavian vein cannula insertion through a supraclavicular approach. This study will compare PIVC and percutaneous subclavian vein cannula insertion through supraclavicular approach, and determine which method is more effective and fast in critically ill patients arriving in the emergency department.

Methods: This prospective, randomized clinical trial involved a total of 98 patients arriving in the emergency department in critical condition. Percutaneous subclavian vein cannula insertion through supraclavicular approach was attempted in 49 patients, and PIVC was attempted in other 49 patients. The timing of cannula insertion and the number of attempts for successful cannulation were compared for the two methods.

Results: Percutaneous subclavian vein cannula insertion through supraclavicular approach was successful in 47 out of 49 patients (96%), and PIVC was successful in 38 out of 49 patients (78%). Average time of percutaneous subclavian vein cannula insertion through supraclavicular approach was 27.7 seconds (range 15-90 seconds), and the average time of PIVC was 68.64 seconds (range 25-150 seconds).

Conclusion: Compared with PIVC, percutaneous subclavian vein cannula insertion through supraclavicular approach is faster and more effective to gain venous access in critically ill patients arriving in emergency department.

## Introduction

Peripheral intravenous cannulation (PIVC) is a universal procedure to get venous access in hospital emergency settings. Venous access is not only useful for sampling blood required for various laboratory tests, but is also critical for the administration of intravenous medications, fluids, chemotherapy, parenteral nutrition, and blood products [1].

Sometimes in emergency department, when one can not get peripheral venous access due to some reason like shock, a central venous access could be established effectively and in short time by percutaneous subclavian vein cannula insertion through supraclavicular approach. This central venous access serves all the purposes a peripheral venous access serves, especially the rapid infusion of fluids (via large bore cannulas) needed in emergency settings. This study will compare the subclavian and peripheral IV cannula insertion to determine which method is more effective and faster in critically ill patients arriving in the emergency department.

Moreover this central venous access via cannula can be converted into a central venous line by Seldinger technique that will have additional benefits of increased line security in situ, increased longevity, less infection, larger and multiple lumens for more rapid administration of multiple drugs and fluids at one time, a route for total parenteral nutrition and central venous pressure monitoring.

The infraclavicular approach of subclavian vein catheterization has become an established technique after it was first described by Aubaniac in 1952 [2]. An alternate supraclavicular approach of subclavian vein catheterization was first described by Yoffa in 1965 [3]. However, it is used less often than the old ‘conventional’ infraclavicular approach. Although there are no randomized trials, the best evidence shows that the supraclavicular approach is more advantageous than the infraclavicular approach of subclavian vein catheterization in a number of aspects.

The incidence of complications like pneumothorax, arterial puncture, hemothorax, brachial plexus, or other nerve injuries, catheter malposition, thrombosis, or thrombophlebitis is zero with percutaneous subclavian vein cannula insertion through the supraclavicular approach.

## Materials and methods

This prospective, randomized clinical trial involved a total of 98 patients arriving in the emergency department of Combined Military Hospital (CMH), Lahore, Pakistan, in a critical condition from January 2017 to March 2017.

A total of six registrars were trained in both peripheral and subclavian IV cannula insertion through supraclavicular approach [4,5]. They were divided into two groups: group A and group B, with three members in each group. They were rotated such that one member from each group was present at a time in the emergency department. One was assigned to pass a 3cm long 18G cannula into a peripheral vein, and the other was assigned to pass a similar 3cm long 18G cannula into the subclavian vein through the supraclavicular approach. Both groups had to be assigned both approaches alternatively.

In supraclavicular approach (Garcia et al. technique), the needle prick was done 1cm lateral to the lateral head of the sternocleidomastoid muscle and 1cm posterior to the clavicle and directed at a 5-degree angle from the coronal plane, 50 degrees from the sagittal plane and 40 degrees from the transverse plane [5].

Timing of cannula insertion was defined as the time from skin-puncture by the needle to the appearance of blood in the hub of the cannula. A third observer observed the timing. Failure was defined as >3 attempts or more than three minutes required for the cannula insertion. Success was defined as maintenance of flow by IV infusion for more than one hour.

The subclavian cannula was later on stitched, and all of them were later on converted into central venous lines by the Seldinger technique from 1-8 hours after cannula insertion.

The Chi-Square test was used to assess the relationship between subclavian and peripheral IV cannulation success rate. P-values less than 0.05 indicated statistical significance. All statistical calculations were performed using SPSS version 20 software.

## Results

**Figure 1:**
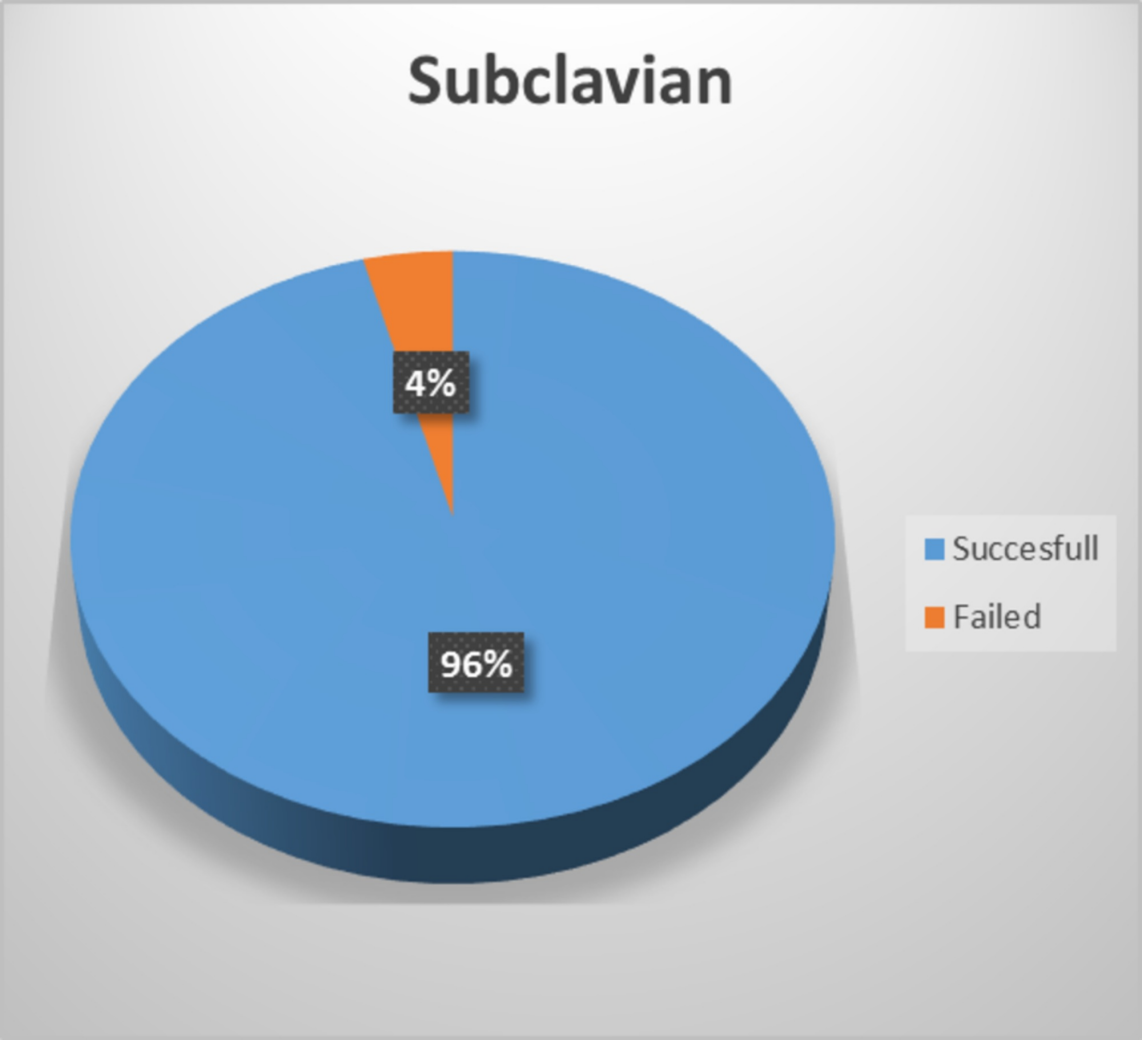
Percentage success of percutaneous subclavian vein cannula insertion through supraclavicular approach

**Figure 2:**
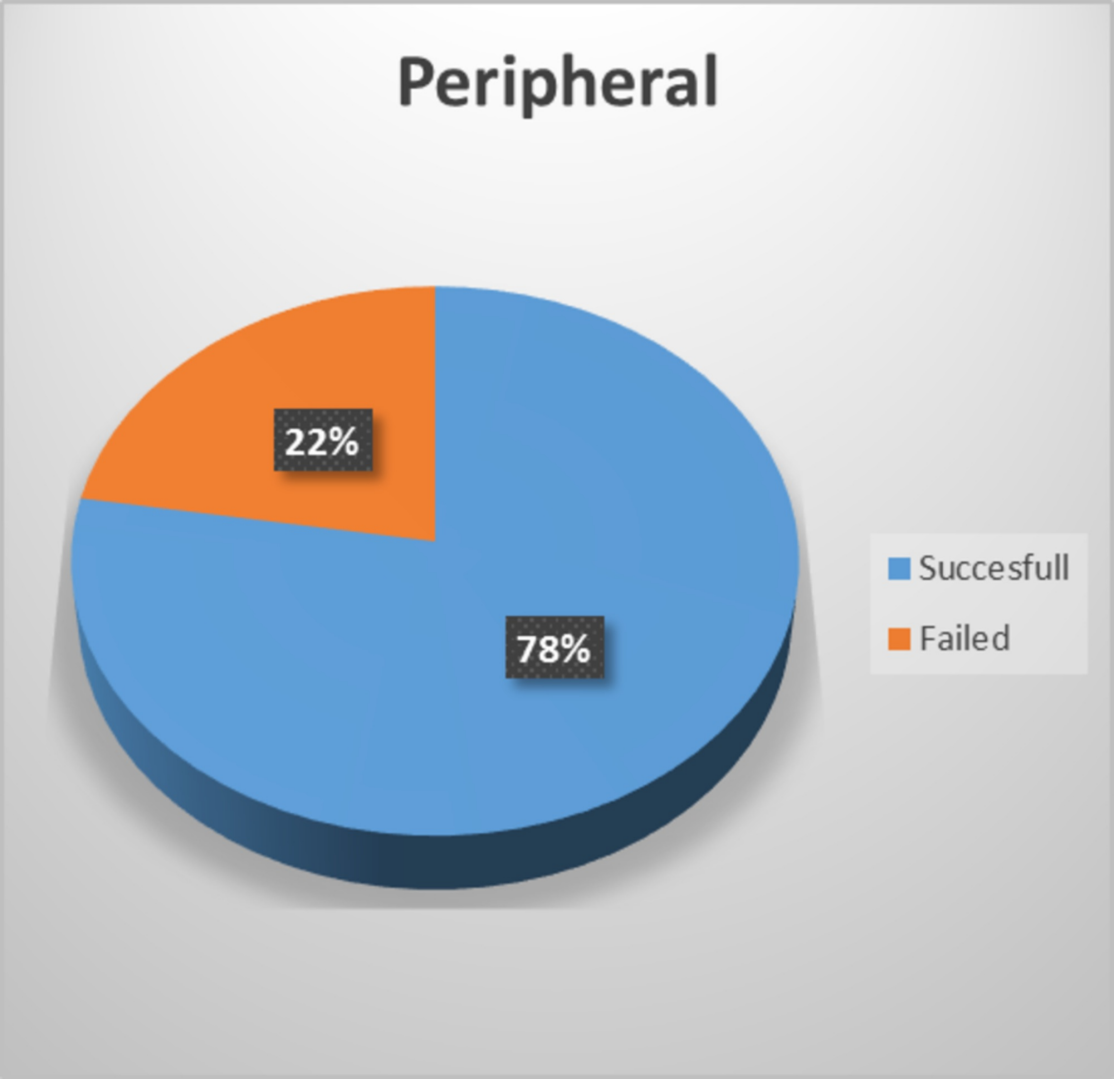
Percentage success of peripheral IV cannula insertion

The study included a total of 98 patients, with percutaneous subclavian vein cannula insertion through a supraclavicular approach attempted in 49 patients and peripheral IV cannula insertion attempted in the other 49 patients. Percutaneous subclavian vein cannula insertion through supraclavicular approach was successful in 47 patients (96%) (Figure 1), and peripheral IV cannula insertion was successful in 38 patients (78%) (Figure 2).

**Figure 3:**
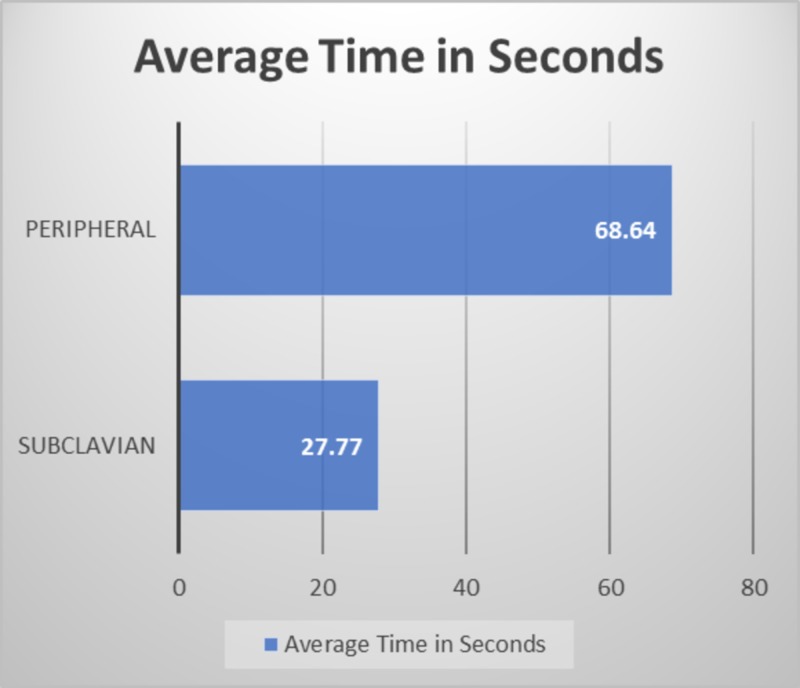
Average time of percutaneous subclavian vein cannula insertion through supraclavicular approach versus average time of peripheral IV cannula insertion

Average time of percutaneous subclavian vein cannula insertion through supraclavicular approach was 27.7 seconds (range: 15-90 seconds) and average time of peripheral IV cannula insertion was 68.64 seconds (range: 25-150 seconds) (Figure 3).

The average number of attempts in successful percutaneous subclavian vein cannula insertion through the supraclavicular approach was 1.36, and in successful peripheral IV cannula insertion was 1.86. Percutaneous subclavian vein cannula insertion through the supraclavicular approach was successful in 33 patients (67%) in the first attempt, and 11 patients (22%) in the second attempt. Peripheral IV cannula insertion was successful in 13 patients (27%) in the first attempt, 19 patients (39%) in the second attempt.

Prior to running analyses, the assumption of normality was assessed by visualizing the data through histograms. It was also supplemented with statistical tests such as Kolmogorov-Smirnov and Shapiro-Wilk test.

Mann Whitney U test was run to analyze the difference in time taken (mean rank) by percutaneous subclavian vein cannula insertion and PIVC in critically ill patients. The difference in the number of attempts for a successful cannulation (mean rank) by the two methods was also assessed with this test.

**Figure 4:**
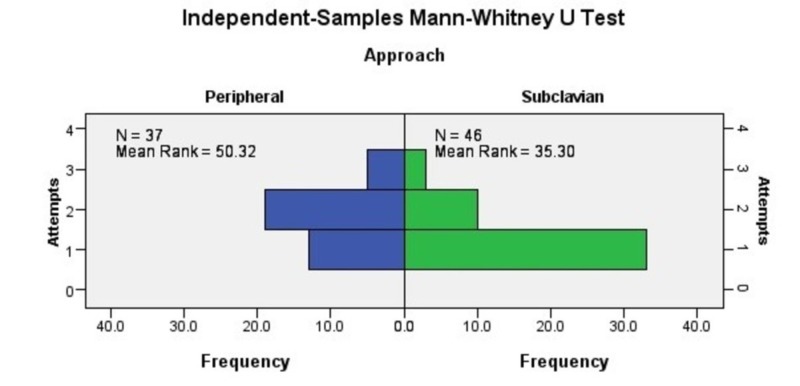
Difference in number of attempts for a successful cannulation by the two methods

Independent sample Mann Whitney U test revealed that successful percutaneous subclavian vein cannula insertion through supraclavicular approach required less number of attempts than successful PIVC (standardized test statistic= 3.182, P < .001, n=83). Detailed results have been presented in Figure 4.

**Figure 5:**
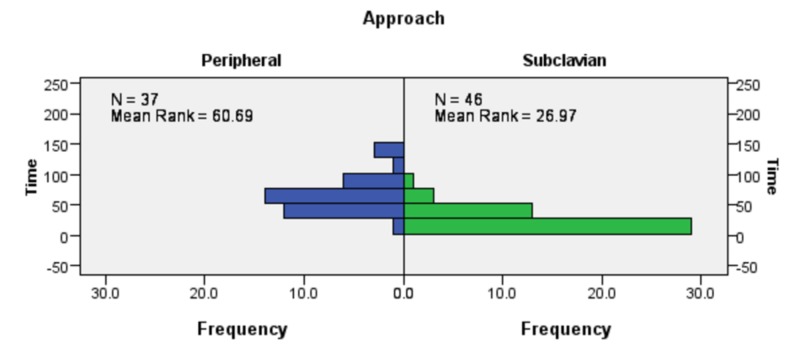
Difference in time taken by percutaneous subclavian vein cannula insertion and PIVC

Independent sample Mann Whitney U test revealed that percutaneous subclavian vein cannula insertion through supraclavicular approach required less time than PIVC for a successful cannulation (statistic= 6.391, P < .001, n= 83). Detailed results have been presented in Figure 5.

## Discussion

PIVC is the most common procedure to get venous access in hospitals, and its omnipresence was shown in a point prevalence study done in a European hospital; over 84% of patients had a vascular access device of some type, with 80% of these PIVCs [6].

Occasionally PIVC fails, and the failure rate is greater in critically ill patients arriving in emergency departments. Risk factors for PIVC failure have been found out in large prospective studies, and prevention of PIVC insertion failure is increasing with the use of specialist teams [7-9].

Successful PIVC is more likely if performed by clinicians who are more experienced in this procedure and have an increased perception of the success probability. Some patient factors also increase the chances of successful cannulation, e.g., 'normal' body weight, visible vein/s, and cubital fossa placement [10].

When PIVC fails, usually central venous access is achieved via catheterization. Moayedi et al. successfully inserted a catheter into the internal jugular vein in patients with difficult intravenous access in 88% of cases, with a mean time of 4.4 min. The only complication was the loss of patency that occurred in 14% of cases [11]. In our study, we got central venous access into the subclavian vein through a supraclavicular approach with a 3cm long 18G cannula successfully in 96% of cases with a mean time of 27.7 seconds.

There are many anatomic advantages of the subclavian vein for the venous access that include its large diameter, absence of valves, and ability to remain patent and in a relatively constant position [3,12]. Subclavian vein catheterization has a lower risk of thrombosis and catheter-related infection than femoral or internal jugular vein catheterization [13]. In our study, the incidence of complications like pneumothorax, arterial puncture, hemothorax, brachial plexus or other nerve injuries, catheter malposition, thrombosis or thrombophlebitis is zero with percutaneous subclavian vein cannula insertion through the supraclavicular approach.

Although ultrasound is a huge progress in the placement of central lines, it is not always available in most of the hospital settings. For the same reason, landmark-based central line placement remains the most prevalent technique. The supraclavicular approach of subclavian vein catheterization is at least as safe as other approaches. Moreover, it is easy to perform, and there are fewer chances of misplacement [14].

Supraclavicular approach of subclavian vein catheterization is superior to infraclavicular approach in a number of aspects: a shorter distance from skin to vein; a larger target area; a well-defined insertion landmark (the clavisternomastoid angle); a straighter path to the superior vena cava; less proximity to the lung; and lesser chances of pleural or arterial puncture [3,12,15-18]. Moreover, the supraclavicular approach less often results in interruption of cardiopulmonary resuscitation (CPR) or tube thoracostomy than the infraclavicular approach [19,20].

Sterner et al. carried out a comparison of the supraclavicular approach and the infraclavicular approach for subclavian vein catheterization [21]. There were 38 failures (15.5%) among 245 patients in the supraclavicular group, with one malposition and five complications. There were 51 failures (20.0%) among 255 patients in the infraclavicular group, with 21 malpositions and 13 complications. No differences were significant except that of malpositions (P less than .01). Analysis of this comparison study reveals that both the infraclavicular and supraclavicular approaches to subclavian vein catheterization are good choices, with little difference in success rate or complications. Using the other approach, if the initial approach was unsuccessful, resulted in a high overall success rate and a low overall complication rate.

A randomized prospective study by Dronen et al. compared the supraclavicular and infraclavicular approaches in 76 patients undergoing CPR [19]. Forty-four supraclavicular attempts and 45 infraclavicular attempts were analyzed. Successful cannulation rates with the two approaches were comparable (90% with the supraclavicular approach and 84% with the infraclavicular approach, p>0.05). The mean number of needle sticks required for the cannulation was similar with the two approaches. However, the incidence of catheter malpositioning or kinking was significantly greater with the infraclavicular approach (26% versus 7% with the supraclavicular approach, p<0.05). Moreover, undue CPR interruption (for ≥ 5 seconds) occurred in 20% of supraclavicular attempts and 40% of infraclavicular attempts (p<0.025). No major complications were noted with both approaches. Based on this study, the subclavian vein catheterization via the supraclavicular approach is reasonably superior to the infraclavicular approach when central venous access is required during CPR.

Patrick et al. showed the success of the supraclavicular approach using Yoffa’s original technique (success rate, 97.7%) as well as modifications to patient position, landmarks, and angles (success rate, 98.6%).

## Conclusions

Compared with peripheral IV cannula insertion, percutaneous subclavian vein cannula insertion through the supraclavicular approach is faster and more effective in gaining venous access in critically ill patients arriving in the emergency department*.*
